# Health-seeking pathway of drug-resistant TB patients in Vadodara, India

**DOI:** 10.5588/pha.23.0019

**Published:** 2023-12-07

**Authors:** M. Sheth, K. Shringarpure, B. Modi, R. Damor, L. Manikam

**Affiliations:** 1 Department of Community Medicine, GCS Medical College, Hospital and Research Centre, Ahmedabad, India; 2 Department of Community Medicine, Medical College Baroda, Vadodara, India; 3 Aceso Global Health Consultants Pte Limited, Singapore, Singapore; 4 Department of Community and Family Medicine, All India Institute of Medical Sciences, Rajkot, India; 5 Department of Epidemiology and Public Health, Institute of Epidemiology and Health Care, University College London (UCL), London, United Kingdom

## Abstract

**BACKGROUND:**

Health-seeking behaviour refers to patients’ choices regarding their preferred healthcare destination and the timing of seeking assistance for treatment. Patients with TB usually first approach the private sector and/or lose several months’ time in inappropriate diagnosis and treatment due to lack of awareness regarding the availability of standard treatment protocols. This can lead to poor outcomes such as drug-resistant TB (DR-TB) and/or death.

**METHODOLOGY:**

A cross-sectional study was conducted to examine the health-seeking pathway and delays in diagnosis and initiation of DR-TB treatment among patients registered with the DR-TB centre in Vadodara District (India).

**RESULTS:**

A total of 93 patients were enrolled in the study; the median age was 35 years (IQR 24–45). For the first visit, 59 (63%) patients chose a public healthcare facility, mainly because the facility was near their residence (*n* = 20, 21.5%). The median delay in reaching the first healthcare facility was 12 days (IQR 7.5–30). Delay in reaching second- and third-level care was respectively 25 days (IQR 9–68) and 16 days (IQR 4–67).

**CONCLUSION:**

Two-thirds of patients required visits to a second healthcare centre for diagnosis, while one third needed a third visit. The overall median delay for reaching the DR-TB centre was 60 days (IQR 26–122). The median duration from symptom onset to the first healthcare contact fell within the timeframe for screening symptoms in standard diagnosis.

## Abstract (fr)

**CONTEXTE:**

Le comportement de recherche de santé fait référence aux choix des patients concernant leur destination préférée en matière de soins de santé et le moment où ils recherchent de l’aide pour le traitement. Les patients atteints de TB s’adressent généralement en premier lieu au secteur privé et/ou perdent plusieurs mois en diagnostics et traitements inappropriés en raison d’un manque d’information sur la disponibilité des protocoles de traitement standard. Cela peut conduire à des résultats médiocres tels que la TB résistante aux médicaments (DR-TB, pour l’anglais « drug-resistant TB ») et/ou la mort.

**MÉTHODOLOGIE:**

Une étude transversale a été menée pour examiner le parcours de recherche de soins et les retards dans le diagnostic et l’initiation du traitement de la DR-TB parmi les patients enregistrés au centre de DR-TB dans le district de Vadodara (Inde).

**RÉSULTATS:**

Au total, 93 patients ont participé à l’étude ; l’âge médian était de 35 ans (IQR 24–45). Pour la première visite, 59 patients (63%) ont choisi un établissement de santé public, principalement parce que l’établissement était proche de leur résidence (*n* = 20 ; 21,5%). Le délai médian pour atteindre le premier établissement de santé était de 12 jours (IQR 7,5–30). Le délai pour atteindre le deuxième et le troisième niveau de soins était respectivement de 25 jours (IQR 9–68) et de 16 jours (IQR 4–67).

**CONCLUSION:**

Deux tiers des patients ont dû se rendre dans un deuxième centre de soins pour obtenir un diagnostic, tandis qu’un tiers a dû se rendre dans un troisième centre. Le délai médian global pour atteindre le centre de lutte contre la TB était de 60 jours (IQR 26–122). La durée médiane entre l’apparition des symptômes et le premier contact avec les soins de santé correspondait au délai de dépistage des symptômes dans le cadre du diagnostic standard.

TB is a continuing public health problem in India (incidence: 188/100,000 population). Moreover, India has the highest burden of multidrug-resistant TB (MDR-TB) cases in the world. The estimated number of MDR-TB and extensively drug-resistant TB (XDR-TB) cases were respectively 4 and 1/100,000 according to the 2021 WHO Global TB Report.^1^ The first National Drug Resistance Survey revealed that nearly 28% of TB patients were resistant to any anti-TB drug (22% among new and 36.8% among previously treated patients) and 6.2% were MDR-TB (2.8% among new patients and 11.6% among previously treated patients).^2^ TB control has been ongoing in the country for more than 50 years. The Revised National TB Control Programme (RNTCP) has been renamed the National Tuberculosis Elimination Programme (NTEP). Under this programme, the 2017–2025 National Strategic Plan (NSP) was launched with a four-pillar strategy to ‘Detect-Treat-Prevent-Build’.^3^ The first and second pillar emphasises on early detection of TB and treatment initiation. Delay in diagnosis, inadequate treatment, drug resistance are important factors contributing to the TB burden.

Delay in diagnosis and the subsequent delayed treatment initiation can lead to greater DR-TB resistance, as well as increased mortality among TB patients. Delay in the diagnosis and initiation of treatment could be attributed to health-seeking behaviour of the patient and delay in diagnosis from the provider’s side.^4^

Health-seeking behaviour for TB can be described as patients’ preference for the healthcare facility and the timing of seeking treatment according to their own choices.^5^ Patient or family behaviour is typically shaped by their perception of the disease, and their response is influenced by the availability of healthcare services that help save time, money and effort.^6^ This type of behaviour can lead to delays in diagnosis and treatment. Chakravarty et al. conducted a study that identified a convoluted patient pathway, with none of the patients originating from the same geographical area. These patients selected healthcare facilities based on their convenience, which may or may not have been part of the integrated healthcare system.^7^

Regarding the provider delay documented by Kelkar-Khambete et al., the evidence indicates that patients had to visit multiple healthcare facilities due to the limitations of each facility in terms of diagnosis and available treatment.^8^ The median delay in reaching the first healthcare facility was found to be 30 days (interquartile range [IQR] 21–60) and overall median delay in initiating treatment was 237 days (IQR 109–491).^9^ Another contributing factor to delay in the Indian context is the practice of initiating DR-TB treatment at the DR-TB centre, typically located in a tertiary care hospital associated with a medical college. The distance to these centres may vary for each patient, with longer distances hindering travel and decision-making, resulting in delays.^10^ Studies conducted in Mumbai, Delhi, and South India by Chakravarty et al. and Rathi et al. have examined the health-seeking pathways, but none have specifically investigated delays in diagnosis and treatment initiation.^7^,^9^ Our study is the first to explore both the health-seeking pathway and delays in Western India, Central Gujarat. The knowledge gained from this research can help bridge the gap between symptom identification and early disease diagnosis, as well as between disease identification and the initiation of early treatment. Therefore, we conducted this study with the objective of investigating the health-seeking pathway and delays in the diagnosis and initiation of DR-TB treatment for patients seeking care at the DR-TB Centre in Vadodara District, affiliated with the tertiary hospital of a medical college.

## METHODOLOGY

### Study design, study population and sample size

A cross-sectional study was carried out among patients with DR-TB (including all five categories mentioned under the operational definitions below) registered with the DR-TB Centre, Vadodara District.

In India, as per the programmatic guidelines for TB elimination, the government established DR-TB Centres, with the aim of having one in each district.^11^ Patients diagnosed with DR-TB initially visit these centres for pre-treatment evaluation and counselling, after which their treatment begins. All patients who were enrolled at the DR-TB Centre, Vadodara District, between January 2021 and September 2021, were included in this study. The study duration was extended by six months due to a reduced number of patient registrations during the COVID-19 lockdown. Because of the lockdown, many patients couldn’t be met in person; therefore, they were interviewed over the phone. In cases of non-response, three attempts were made to reach the patients by phone. Out of these attempts, 40 patients did not respond, while 12 had provided incorrect contact details. Of a total 162 patients registered during the study period, 93 patients were contacted and gave consent for the study. Of these, 7 (7.5%) were below 18 years of age. We interviewed the parents/guardians related to the health pathway and delays in diagnosis in case of children less than 18 (as per programmatic guidelines) as they could be a credible source of information.

### Study setting

According to the recent PMDT guidelines, patients are required to register themselves at a DR-TB centre for pre-treatment evaluation before commencing DR-TB treatment.^11^ Typically, these DR-TB centres are located within medical colleges. After the patients undergo evaluation at the DR-TB centre, their treatment regimen is determined, and treatment is initiated following counselling. This study was conducted at the DR-TB Centre in Vadodara District, which serves patients from eight different districts. Patients are referred to this centre for baseline drug sensitivity testing, pre-treatment evaluation, treatment initiation, and counselling regarding treatment and potential adverse drug reactions.^11^

### Data variables

Exposure variable such as sociodemographic details of the patients, symptoms, first healthcare facility contacted and outcome of the disease were collected. Questions pertaining to the pathway prior to reaching our DR-TB centre (including treatment access, how, who, where, when and time at different health facilities) before diagnosis and treatment inititaion were assessed to aid in pathway analysis.

### Data collection and analysis

Contact details of the patients were obtained from patients records after due permissions. Data were collected using a pre-tested semi-structured questionnaire in a single telephone interview. A second call was made in case of incomplete information. Data were entered in MS Excel (Microsoft, Redmond, WA, USA), and the health-seeking pathway with median duration and delay in diagnosis and overall treatment initiation were presented in graphs. Patient-side delay was determined by assessing the time patients took to reach the initial healthcare facility, while provider-side delay was calculated based on the time taken to initiate treatment after arriving at the first healthcare facility. Interviews were conducted within 21 days of treatment initiation to rule out recall bias.

### Ethical consideration

The study was conducted after obtaining permission from The Institutional Ethics Committee for Human Research (IECHR) and the NTEP. Patients were contacted and telephone consent for the interview was obtained; if patients were not available at the time of the interview, the telephone interviews were conducted at their convenience.

### Data confidentiality

Interviews were conducted after ensuring due confidentiality. Data were entered in a pre-designed data capture format based on the information recorded in the registers. As unique identification numbers were assigned to patients, no names were mentioned and confidentiality was maintained. Hard copies of data were kept under lock and key in a cupboard designated for this specific research, while the electronic records were secured using password-protected files, allowing limited access to soft copies and hard copies. The Principal Investigator was personally responsible for data collection; these data were shared with co-investigators after removing individual identifiers for analysis purposes. Records will be maintained for 5 years after completion of study.

### Specific patient benefits

At the conclusion of each in-depth interview, a simplified information sheet in the local language was presented orally. This sheet provided an overview of MDR-TB, stressed the significance of adhering to the treatment, and informed the participants about the Direct Benefit Transfer and other available support schemes during their treatment.

### Definitions

Presumptive DR-TB refers to individuals eligible for screening for rifampicin (RIF, R) resistance at the time of TB diagnosis or during treatment for drug-susceptible TB (DS-TB). This category also includes those with isoniazid (INH, H) mono/poly DR-TB. It comprises all reported TB patients, whether from the public or private sector, follow-up cases with positive microscopy results, including those who have experienced treatment failure with standard first-line treatment and those on the INH mono/poly DR-TB regimen. It also covers clinical non-responders, including paediatric non-responders.^3^ Isoniazid-resistant TB are patients with resistance to INH but susceptibility to RIF.^3^ Mono-resistant TB are patients with TB who are resistant to a single first-line anti-TB drug.^3^ MDR-TB refers to patients with resistance to both INH and RIF, either alone or in combination with resistance to other first-line anti-TB drugs. MDR-TB patients may also have additional resistance to fluoroquinolones (FQs) or other anti-TB drugs.^3^ Poly-drug resistant TB (PDR-TB) refers to patients who are resistant to more than one first-line anti-TB drug, excluding both INH and RIF.^3^ RIF-resistant TB (RR-TB) include patients who are resistant to RIF, as determined using phenotypic or genotypic methods. This category includes any form of RIF resistance, such as mono-resistance, poly-resistance, multidrug resistance (i.e., MDR-TB) or extensively drug-resistant TB (XDR-TB).^3^ For programmatic purposes in India, a child is an individual up to and including 18 years of age, which also covers adolescents aged 10–18 years.^12^

## RESULTS

Out of the total of 162 registered patients, 93 could be successfully contacted. This study included a total of 93 newly diagnosed DR-TB patients, with a mean age of 34.5 years (standard deviation [SD] ±12.96). The majority of these patients (*n* = 90, 96.8%) had pulmonary TB. The study comprised 55 (59.1%) males, and the majority (*n* = 83, 89.3%) were identified as Hindu. Furthermore, 79 (84.9%) of the patients had received some level of education, and 65 (69.9%) resided in urban areas. The median delay for seeking care did not differ significantly across various parameters, including age groups, sex, religion, educational level, occupation, socio-economic status and residence (*P* > 0.05) (Table 1). To note, the primary symptoms that led patients to seek healthcare were cough, reported by 70 (75%) individuals, and fever, which prompted 31 (34%) patients to seek medical attention. A smaller number of patients sought treatment due to breathlessness, with only 10 (11%) experiencing this symptom.

**TABLE 1:** Socio-demographic details of the patients

Variable	Frequency *n* (%)	Total delay Median [IQR]	*P*-value*
Age group, years			0.60
<18	7 (7.5)	56 [4–72]	
19–25	25 (26.9)	66 [34–122]	
26–35	20 (21.5)	59 [15.5–104.5]	
36–45	20 (21.5)	72.5 [26–180.5]	
46–55	14 (15.1)	38.5 [22–95]	
56–65	7 (07.5)	62 [31–728]	
Sex			0.77
Male	55 (59.1)	59 [25–127]	
Female	38 (40.9)	61.5 [28–95]	
Religion			0.95
Hindu	83 (89.3)	60 [25–122]	
Muslim	09 (09.7)	72 [31–74]	
Christian	01 (01.1)	50 [50–50]	
Education status			0.97
Illiterate	14 (15.1)	60.5 [31–93]	
Literate	79 (84.9)	60 [22–127]	
Occupation			0.08
Daily wages	23 (24.7)	84 [36–177]	
Job	16 (17.2)	43 [12.5–114.5]	
Business	04 (04.3)	160.5 [104.5–261]	
Student	07 (07.5)	56 [20–72]	
Home maker	27 (29.0)	47 [21–95]	
Unemployed	16 (17.2)	40 [24.5–80.5]	
Socio-economic status			0.97
Above the poverty line	43 (46.2)	61 [22–118]	
Below the poverty line	50 (53.8)	59.5 [27–122]	
Area of residence			0.44
Rural	65 (69.9)	59 [28–102]	
Urban	28 (30.1)	68 [18.5–152]	
Total	93 (100)		

*Mann-Whitney *U*-test *P* < 0.05 considered statistically significant.

### Health-seeking pathway

The health facilities frequented by DR-TB patients, illustrating their healthcare-seeking journey, were categorised into four main groups. Of the 93 patients, 16 received a diagnosis and commenced treatment without being directed to another facility as they initially visited the DR-TB centre as their primary point of care. In our study, patients consulted with as many as four different levels/centres before receiving a diagnosis and starting treatment. Forty-eight patients required only one referral to reach the point of diagnosis and treatment initiation. Twenty-two patients (23%) underwent two referrals. The majority of patients (86/93) received their diagnosis and started treatment at the second referral level. Five patients needed a third referral, while two required four referrals before their DR-TB diagnosis and treatment initiation.

### Reaching the first healthcare setup

Among the total of 93 patients, 77 (82%) opted to visit a healthcare facility of any type (public or private). The remaining patients favoured home remedies and Ayurvedic preparations, as well as obtaining direct medications from a pharmacy, which accounted for 10 (11%) and were screened by field workers, totalling 6 (7%).

For their initial visit, 63% of patients had chosen public healthcare facilities, including primary healthcare centres/urban primary healthcare centres (PHCs/UPHCs) (*n* = 23), community healthcare centres (CHCs) (*n* = 5), district hospitals (*n* = 15) and tertiary care hospitals associated with medical colleges (*n* = 16). The most common reasons for selecting a public healthcare facility for diagnosis and treatment were its proximity to their residence (*n* = 20, 21.5%), previous experience with treatment at a public health facility (*n* = 10, 10.8%) and affordable costs (*n* = 5, 5.4%).

**FIGURE 1:**
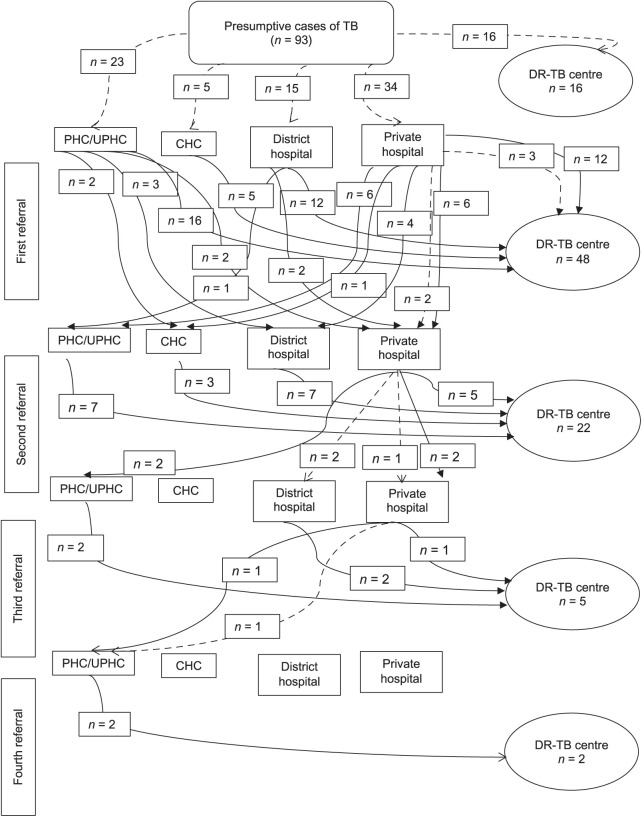
Health-seeking pathway of DR-TB patients. Figure 1 shows health-seeking pathway of the patients in terms of how much health facilities they visited before diagnosis and initiation of the treatment. The health facilities visited by the DR-TB patients, depicting the health-seeking pathway were divided mainly in four categories. DR-TB = drug-resistant TB; PHC/UPHC = primary healthcare centres/urban primary healthcare centres; CHC community healthcare centres.

**FIGURE 2:**
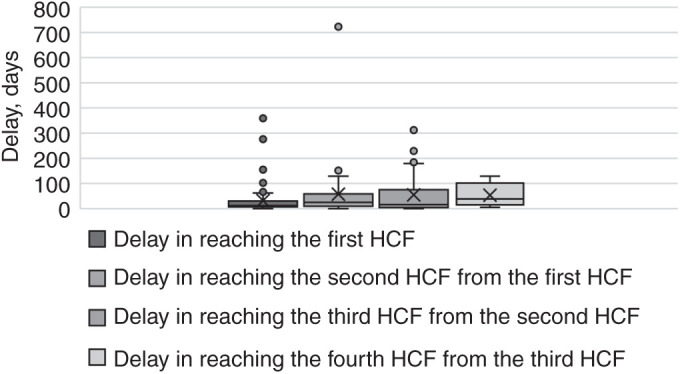
Delay in reaching a HCF. HCF = healthcare facility.

Reasons for choosing a private healthcare facility included its proximity to their residence (*n* = 7, 7.5%), the facility being that of their family physician (*n* = 3, 3.2%) and the expectation of better healthcare services (*n* = 3, 3.2%) (Table 2). The median delay in reaching the first healthcare facility from the patient’s side was 12 days, with an interquartile range (IQR) of 7.5 to 30 days. ­Figures 1 and 2 show the progression of the healthcare pathway, referrals, and the duration of the delay in diagnosis and treatment initiation following the onset of symptoms for each patient.

**TABLE 2:** Reasons for selecting public healthcare setup

	*n* (%)
Reason for selecting public healthcare setup*	
Nearer to residence	20 (21.5)
Previous history of TB and previous treatment from Government setup	10 (10.8)
Affordable	5 (5.4)
Only hospital in village	4 (4.3)
Prior treatment from same hospital	4 (4.3)
Heard from family and friends	3 (3.2)
Other (Government setup, referred by field worker, NGO, severe symptoms, staff in hospital)	7 (7.5)
Reasons for selecting private setup	
Nearer to residence	7 (7.5)
Family physician	3 (3.2)
For better healthcare	3 (3.2)
Due to COVID	2 (2.2)
Other	4 (4.3)

* Multiple answers possible.

NGO = non-governmental organisation.

### Reasons for referral

The reasons for first referral (*n* = 77) were for specific treatment of DR-TB (*n* = 36, 46.8%), for treatment of TB (*n* = 17, 22.1%), lack of improvement of symptoms (*n* = 13, 16.9%) and treatment not affordable (*n* = 7, 9.1%). Reason for referral to second (*n* = 32) and third (*n* = 7) healthcare facility/setup were also similar; for specific treatment of DR-TB (respectively* n* = 16, 50.0% and *n* = 3, 48.9%), lack of improvement of symptoms (respectively* n* = 6, 18.8% and *n* = 2, 28.6%) and treatment not affordable (*n* = 4, 12.5% and *n* = 2, 28.6%) The reasons for referral are mentioned in Table 3.

**TABLE 3:** Reasons for referral at various levels

Reasons	*n* (%)
Reason for referral from first-level setup to second-level setup (*n* = 77)	
Referred for DR-TB treatment	36 (46.8)
For treatment of TB	17 (22.1)
No improvement of symptoms	13 (16.9)
Treatment not affordable	7 (9.1)
Other (worsening of symptoms wanted a second opinion)	4 (5.2)
Reason for referral from second-level setup to third-level setup (*n* = 32)	
Referred for DR-TB treatment	16 (50.0)
No improvement of symptoms	6 (18.8)
Treatment not affordable	4 (12.5)
Other	3 (9.4)
Reason for referral from second-level setup to third-level setup (*n* = 7)	
Referred for DR-TB treatment	3 (48.9)
Treatment not affordable	2 (28.6)
No improvement of symptoms and worsening condition	2 (28.6)

DR-TB = drug-resistant TB.

### Delay in reaching healthcare facility

The overall median delay of 60 days (IQR 26–122) for patients to reach the DR-TB centre and commence treatment. Specifically, the delay in reaching the second level was 25 days (IQR 9–68) and for the third level, it was 16 days (IQR 4–67). The cumulative delay from the providers’ side amounted to 31 days (IQR 5–75). Figure 2 shows the median delay in days for reaching specific referral centres, while Tables 1 and 4 present the factors influencing the delay in accessing healthcare facilities.

**TABLE 4:** Delays at different health setting

Variable	*n* (%)	Median [IQR]	*P*-value
First healthcare contact			0.029*
Public	59	47 [15–95]	
Private	34	72 [34–151]	
Total number of health set-up visited			0.028†
1	16 (17.2)	21.5 [8.5–94]	
2	48 (51.6)	54.5 [23.5–103]	
3	22 (23.7)	63 [47–95]	
4	07 (7.5)	231 [107–353]	

*Mann-Whitney *U*-test.

^†^Kruskal-Wallis test *P* < 0.05 considered as statistically significant.

## DISCUSSION

DR-TB represents a dual challenge as it impacts patient treatment duration, disease transmission,^13^ and healthcare systems by elevating adverse outcomes and treatment costs.^9^ Despite the significance of this issue, there has been limited exploration of health-seeking patterns and diagnostic and treatment delays within the Indian context.^14^,^15^ Additionally, no studies have focused on Western India and Central Gujarat.

Our study aimed to unveil the health-seeking trajectories and the delays in obtaining diagnoses and DR-TB treatment among patients who visited the DR-TB centre affiliated with a medical college’s tertiary care hospital. An improved understanding of the pathways taken by DR-TB patients could pave the way for enhanced care access, ultimately reducing both delays and treatment expenses.

In our investigation, it was observed that a majority of patients, two-thirds, required a visit to a second-level healthcare facility before receiving a diagnosis and commencing DR-TB treatment. For a small number of patients, five in total, a visit to a third-level healthcare facility was necessary, and just two patients had to access a fourth-level healthcare facility. Notably, 16 patients were diagnosed and started treatment during their first healthcare encounter, possibly due to direct referral by healthcare workers or their prior knowledge of the diagnostic and treatment process. This prior knowledge might have resulted from knowing individuals with TB within their social circles or having previous experience with TB treatment, although this aspect was not explored in depth in our study. Describing the health pathways of patients with previous TB experience could be a valuable avenue for future research.

The overall median delay was 60 days (IQR 26–122) before treatment initiation, and a median delay of 12 days (IQR 7.5–30) in reaching the first healthcare facility from the patient’s perspective. Other studies conducted in India reported different figures, with Rathi et al. finding a median delay of 30 days for public health facilities and 20 days for private health facilities,^9^ whereas Yasobant et al. reported a delay of 12 days.^16^

The substantial delay in treatment initiation suggests a lack of knowledge or prompt referral services for diagnosis and treatment initiation.^16^,^17^ This may also be attributed to patients moving between various healthcare facilities, leading to visits to multiple referral centres.^18^ This is supported by the reasons obtained in our study, including referrals for specific DR-TB treatment, lack of symptom improvement at a particular healthcare facility, treatment affordability issues, and, in some cases, symptom deterioration.

We found that only 16% of patients initiated treatment at the DR-TB centre during their first visit, having gone directly to the district DR-TB centre, while the majority of patients required two or more referrals. A study conducted in Ghana by Queri et al. in 2014 also reported that more than half of the patients began treatment at facilities other than DOTS centres.^19^ There is limited literature available on referrals in DR-TB patients.^9^ A systematic review by Samal in 2016 regarding health-seeking behaviour among TB patients revealed that, on average, 48% of patients sought care at private healthcare facilities for their first point of contact, which is higher than the 37% found in our study.^20^ The primary reasons for selecting the initial contact centre were accessibility, reduced waiting times and familiarity.

Multiple referrals were primarily linked to treatment affordability issues, and there was a significant difference in the median delay in treatment between patients who visited public healthcare facilities and those who chose private facilities. Private facilities are often associated with higher care costs, leading to referrals to other healthcare facilities. Despite the national health programme’s aim of zero catastrophic costs for TB treatment and a standard of care in public healthcare facilities, there may be a lack of awareness about these provisions, resulting in patients seeking care in the private sector. Studies have also indicated that patients prefer private facilities due to the fear of being stigmatised as having TB.^16^

The median delay from the providers’ side in our study was 31 days (IQR 5–75). A systematic review by Getnet et al. in 2017 reported provider-side delays ranging from 2 to 128.5 days (IQR 12–34).^21^ The main reasons for provider-side delays were an overburdened healthcare workforce in TB programmes, inadequate referrals from private practitioners, referral delays, misinterpretation of provisional diagnoses, delayed diagnoses and delayed laboratory reporting.^15^ Another 2016 systematic review by Samal et al. identified key factors contributing to delays in diagnosis and treatment, including a failure to recognise symptoms as severe, work-related pressures, limited access to healthcare facilities, poor socio-economic status, insufficient knowledge about the disease, etc.^20^

Studies conducted in Bangladesh and Kerala, India, reported delays of respectively 68.5 days and 78 days.^4^,^17^ A systematic review by Sreeramareddy et al. identified a median total delay of 55.3 days (IQR 46.5–61.5),^22^ which was slightly lower than our study’s 60-day delay (IQR 26–122), which is more than the standard screening criteria.

These delays may be attributed to factors such as patients’ knowledge of the disease, awareness of available services, self-medication practices, and occupational commitments, as mentioned in other studies.^23^,^24^ Further in-depth qualitative research is necessary to explore the reasons for these delays, both from the patients’ and providers’ perspectives, to better understand the dynamics of the health pathways and the associated delays.

The importance of conducting more studies and analysing health-seeking behaviour cannot be understated, as it will contribute to the design and implementation of patient-centred approaches to TB/DR-TB diagnosis and management. We acknowledge the limitation of not collecting data related to TB contacts and individuals with previous TB episodes. This information could be pertinent and may reduce delays among individuals with previous TB experiences, warranting exploration in future studies.

The primary limitation of our study was our inability to contact all the patients registered within the specified time frame. Given that our study was conducted during the COVID-19 pandemic, the number of patients registered during the designated study period was limited. Consequently, we extended the study duration and conducted interviews over the phone, being careful to collect the required data. Our study focused on questions related to the health pathway before treatment initiation, potentially introducing recall bias. Moreover, our study did not differentiate between diagnostic delays and delays in treatment initiation, which could be examined in future studies.
